# Supplementary material for: Visual Attention Patterns Toward Female Bodies in Anorexia Nervosa—An Eye-Tracking Study with Adolescents and Adults
Source: Behav Sci (Basel). 2025 Jul 29;15(8):1027. doi: 10.3390/bs15081027 (PMC12383188; doi:10.3390/bs15081027)
Supplement: Supplementary file 1 [file behavsci-15-01027-s001.zip › behavsci-3689154-supplementary.pdf]

Table S1. Results of pairwise comparisons for picture category for fixation times of the total sample. ...

Table S2. Total fixation times within the body area of the single weight categories in relation to the area of the bodies (mean ± standard deviation). ....

Table S1: Results of pairwise comparisons for picture category for fixation times of the total sample.

Pairwise comparison	M_{Dif}	95%-CI	p
**Whole body**			
extremely underweight - underweight	.627	.397, .856	<.001
extremely underweight – normal weight	.804	.594, 1.014	<.001
extremely underweight - overweight	2.337	2.129, 2.546	<.001
extremely underweight – extremely overweight	4.050	3.814, 4.286	<.001
underweight – normal weight	.177	-.015, .369	n.s.
underweight - overweight	1.711	1.527, 1.894	<.001
underweight – extremely overweight	3.423	3.214, 3.632	<.001
normal weight - overweight	1.534	1.342, 1.725	<.001
normal weight – extremely overweight	3.246	3.036, 3.456	<.001
overweight – extremely overweight	1.713	1.601, 1.825	<.001
**Unclothed body parts**			
extremely underweight - underweight	-.578	-1.402, .245	n.s.
extremely underweight – normal weight	-.583	-1.704, .539	n.s.
extremely underweight - overweight	1.951	1.121, 2.781	<.001
extremely underweight – extremely overweight	5.460	4.616, 6.305	<.001
underweight – normal weight	-.004	-.995, .978	n.s.
underweight - overweight	2.529	1.681, 3.377	<.001
underweight – extremely overweight	6.038	5.256, 6.819	<.001
normal weight - overweight	2.534	1.624, 3.443	<.001
normal weight – extremely overweight	6.042	5.130, 6.955	<.001
overweight – extremely overweight	3.509	2.863, 4.154	<.001
**WIA**			
extremely underweight - underweight	.573	.287, .859	<.001
extremely underweight – normal weight	188	.189, .566	n.s.
extremely underweight - overweight	2.526	2.174, 2.878	<.001
extremely underweight – extremely overweight	4.618	4.211, 5.025	<.001
underweight – normal weight	-.385	-.692, -.077	.005
underweight - overweight	1.953	1.689, 2.218	<.001
underweight – extremely overweight	4.045	3.742, 4.348	<.001

normal weight - overweight	2.338	2.000, 2.676	<.001
normal weight – extremely overweight	4.430	4.016, 4.844	<.001
overweight – extremely overweight	2.092	1.899, 2.285	<.001

Table S2: Total fixation times within the body area of the single weight categories in relation to the area of the bodies (mean ± standard deviation).

**Category**	**AN patients**	**Control participants**	**p (adolescents vs. adults)**
**extremely underweight**			**.027**
adolescents	10.23 (1.15)	10.38 (.84)	
adults	10.86 (.49)	10.71 (.51)	
**underweight**			**.026**
adolescents	9.71 (.94)	9.72 (.89)	
adults	10.24 (.48)	9.86 (.73)	
**normal weight**			**.053**
adolescents	9.43 (.98)	9.71 (.73)	
adults	9.99 (.44)	9.83 (.52)	
**overweight**			n.s.
adolescents	8.12 (.52)	8.17 (.55)	
adults	8.24 (.31)	8.30 (.34)	
**extremely overweight**			n.s.
adolescents	6.46 (.42)	6.51 (.26)	
adults	6.54 (.22)	6.50 (.24)	
